# ChemTorch: A Deep Learning Framework for Benchmarking and Developing Chemical Reaction Property Prediction Models

**DOI:** 10.1021/acs.jcim.5c02645

**Published:** 2026-02-24

**Authors:** Jasper De Landsheere, Anton Zamyatin, Johannes Karwounopoulos, Esther Heid

**Affiliations:** Institute of Materials Chemistry, 27259 TU Wien, Vienna 1060, Austria

## Abstract

Modeling of chemical reactions is essential for understanding kinetic mechanisms and predicting possible outcomes of reacting systems. Quantum mechanical calculations are accurate but often prohibitively expensive. Deep learning has emerged as a faster alternative, but progress is slowed by a fragmented software ecosystem that hinders reuse, fair comparison, and reproducibility. We present ChemTorch, an open-source framework that streamlines model development, experimentation, hyperparameter tuning, and benchmarking through modular pipelines, standardized configuration, and built-in data splitters for in- and out-of-distribution evaluation. We envision ChemTorch as a foundation for community-driven method development and reproducible benchmarking in chemical reaction modeling. As a first step toward unified benchmarks, we compare four representative modalities for barrier-height prediction on the RDB7 data set, including fingerprint-, sequence-, graph-, and 3D-based approaches. Our results highlight clear advantages of structurally informed models and sharp performance drops under out-of-distribution conditions, highlighting the importance of rigorous benchmarking.

## Graphical abstract

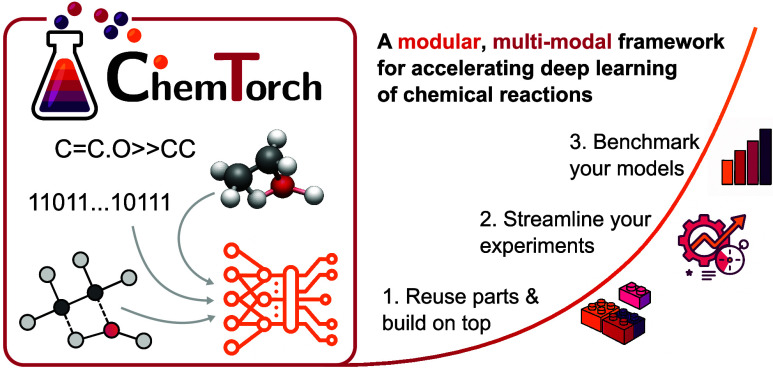

## Introduction

Understanding and predicting how chemical reactions proceed is crucial for applications including synthesis planning, high-throughput experimentation, and process optimization.^1^ Reaction feasibility is typically determined experimentally^2^ or estimated with quantum mechanical (QM) methods that compute activation barriers.^3,4^ While QM methods are considered the gold standard, transition state (TS) searches make them prohibitively expensive, often requiring hours or days per reaction.

Machine learning (ML) offers a complementary alternative by replacing expensive calculations with millisecond-scale inference. Pioneering efforts using kernel ridge regression (KRR) demonstrated the feasibility of predicting reaction barriers with high accuracy and data efficiency.^5–7^ However, deep learning (DL) has since emerged as the dominant paradigm, outperforming traditional methods on large data sets and expanding the scope of ML to a much broader spectrum of reaction tasks.^8^ Regression tasks include activation barriers,^9–13^ enthalpies,^11^ and yields.^14–18^ Classification tasks cover discrete outcomes such as reaction classes.^15,17,19^ Structure-centric tasks focus on predicting transition state geometries^20,21^ or entire reaction pathways.^22^ Finally, conditional tasks include product prediction,^23^ retrosynthesis,^24^ and even multistep route evaluation.^25^

A key design choice in all of these tasks is the reaction representation. Representations explored in the literature include SMILES strings with chemistry-aware tokenization,^16,23,26^ fingerprints,^15,27,28^ graph-based schemes,^9,17,29^ and 3D structures of molecular conformers.^30,31^ Additionally, each representation can be paired with a range of architectures, from classical machine learning and simple multilayer perceptrons (MLPs) to graph neural networks (GNNs), transformers and E(3)-equivariant neural networks (E(3)-NNs) with new representations and models likely to emerge in the future. Representative examples are summarized in Table 1.

**Table 1:** Selected Studies Highlighting the Diversity in Tasks, Representations, and Neural Architectures Explored in Prior Work; the Table is Illustrative Rather than Comprehensive

paper	task	representation	architecture
Probst et al.^15^	reaction classification, reaction yield prediction	DRFP	MLP, xgboost, gradient boost
Yang et al.^32^	molecular property prediction	CGR	D-MPNN
Vadaddi et al.^9^	barrier height prediction	atom mapped reactant and product graphs	D-MPNN,^33^ EGAT^34^
Spiekermann et al.^11^	barrier height prediction	CGR	D-MPNN^33^
Grambow et al.^12^	barrier height prediction	atom mapped reactant and product graphs	D-MPNN^33^
Schwaller et al.^23^	outcome prediction	SMILES tokenization	transformer^35^
Xu et al.^17^	reaction classification, reaction yield prediction	Delta-Mol graphs	hybrid GNN^36^-transformer^35^
Schwaller et al.^16^	reaction yield prediction	SMILES tokenization	BERT^37^
Cao et al.^26^	reaction classification, reaction yield prediction	hierarchical tokenization	HAN^38^
Gasteiger et al.^30^	molecular property prediction	3D graph	E(3)-NN
van Gerwen et al.^13^	barrier height prediction	R & P geometries, optional atom mapping	O(3)-equivariant tensor field network^39^
Galustian et al.^20^	TS prediction	3D coordinates, CGR^29^	flow matching^40^ with E(3)-NN backend^41^

The choice of reaction representation and associated neural architecture is therefore nontrivial, as different representations encode distinct types of chemical information and entail markedly different computational cost. For barrier height prediction, for example, explicit 3D representations of reactant and transition state geometries convey rich information that directly relates to the underlying potential energy surface. In contrast, graph-based representations encode only topological connectivity and must implicitly average over the conformational ensemble associated with the reaction. As shown by Spiekermann et al.,^11^ E(3)-equivariant neural networks can outperform GNNs in barrier-height prediction, but require access to transition state geometries that are costly to obtain in practice. This trade-off has motivated hybrid screening strategies, in which comparatively inexpensive graph-based models are used to preselect promising reactions, followed by more accurate predictions based on semiempirical or quantum-mechanical geometries.^42^ Consequently, researchers have to evaluate whether a representation suits the specific task and if the cost of generating inputs permits practical application.

The diversity of representations and architectures is also reflected in the current software ecosystem with implementations of varying usability and extensibility. As an example of well-documented research code, the repositories of Schwaller and co-workers have popularized transformer-based models for reaction prediction,^23^ yield estimation,^16^ and mapping of chemical reaction spaces.^19^ Packages such as SchNetPack^43^ and NequIP^44^ provide modular backends and reusable components for building equivariant 3D models for interatomic potentials. Further, Chemprop^32,33^ is a widely used package for molecular and reaction property prediction using directed message passing neural networks (D-MPNNs), a special flavor of GNNs. It provides a rich suite of features, including uncertainty quantification, pretraining, transfer learning, hyperparameter optimization, and a convenient command-line interface (CLI). Its success highlights the need for accessible and well-documented frameworks that simplify common research workflows and enable developers to build on top.

While these efforts demonstrate the value of open and user-friendly code, they also highlight important limitations. Most implementations are specialized (tied to a single task, model family, or data modality), which makes it difficult to assess performance across representations. This is exacerbated by the fact that implementations are scattered across different repositories, possibly with subtle differences in data handling and evaluation protocols. Lastly, reaction models are known to be highly sensitive to data set composition and data splitting, with performance varying largely between in-distribution and out-of-distribution test cases.^9,11,13^ These issues underscore the need for a unified framework that reduces engineering overhead but also enables fair and reproducible benchmarking across tasks, representations, and models.

To this end, we present ChemTorch, a general open-source software framework for deep learning of chemical reactions. ChemTorch is built to streamline experimentation and simplify model development. Due to its modular design, ChemTorch is extensible to any task, representation, or model. This first release comes with out-of-the-box implementations of fingerprint-, sequence-, graph-, and 3D-based pipelines for regression and classification. With integrated functionality for hyperparameter optimization, reproducibility, and reaction-specific data splitters, ChemTorch also lays the groundwork for unified and transparent benchmarks in reaction deep learning. We envision ChemTorch as a community platform and welcome contributions. The project is publicly available on GitHub at https://github.com/heid-lab/chemtorch.

## Design Philosophy

In ChemTorch, reaction modeling workflows are formalized as a small set of modular components (Figure 1). Each implements a predefined interface following “design by contract”,^45^ ensuring interoperability while allowing flexible extension. This modularity promotes reusability and extensibility: users can assemble full pipelines or swap out individual parts, modifying only the components relevant to their task. A clear separation of concerns further improves readability, maintainability, and testability of the code. In the following, we describe the key abstractions used by ChemTorch.

**Figure 1:**
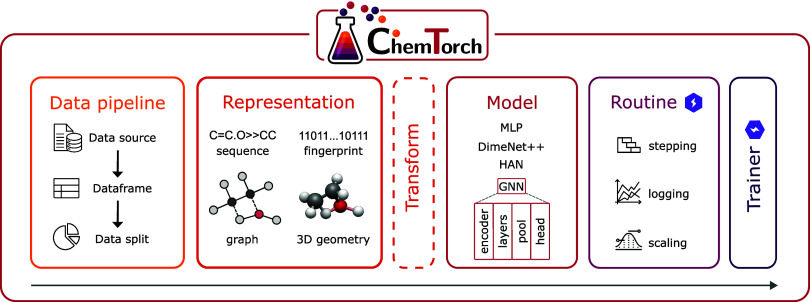
Overview of the ChemTorch pipeline. Reaction modeling workflows are organized into modular abstractions that can be flexibly combined into end-to-end pipelines. The data pipeline ingests, standardizes, and splits the data. Representations encode the chemical reactions (optionally enriched by transforms). The model defines the predictive logic and can also be hierarchically decomposed. The routine defines stepping logic, handles metric logging, and output scaling (in regression). The trainer orchestrates execution across training, validation, and testing. This modular design reduces engineering overhead and enables systematic benchmarking across modalities and architectures.

### Data Pipeline

The data pipeline ingests raw data sets and prepares them for modeling. It ensures that the reaction data is converted into a uniform tabular format and partitioned consistently across training, validation, and test sets. Built-in splitters cover common settings such as random, scaffold, size-, target-, and reaction core-based splits with integrated saving functionality, enabling reproducible in- and out-of-distribution evaluation. Users can extend the pipeline with custom splitters, preprocessing steps, or data sources without affecting downstream components.

ChemTorch comes with ready-to-use pipelines for data sets available in our reaction database repository[Fn fn1], including E2 and SN2 reactions by Von Rudorff et al.,^46^ the [3 + 2] cycloaddition data set by Stuyver et al.,^47^ RGD1,^48^ RDB7,^4^ and the USPTO-1k data set curated by Schwaller et al.^19^ However, users are free to add their own data sets by simply defining a data pipeline configuration reusing the existing components.

### Representation

Representations convert chemical reactions into machine-readable inputs for models. ChemTorch supports fingerprints (e.g., DRFP^15^), token sequences (SMILES,^19^ hierarchical tokenizers^26^), graph-based schemes (e.g., CGR^29^), and 3D structures^49^ (e.g., transition state geometries). By treating representations as modular abstractions, researchers can directly compare the impact of different reaction encodings on predictive accuracy within a common pipeline.

### Transforms

Transforms are optional modules that modify representations before modeling. For graph-based inputs, this may include adding positional or structural graph encodings or inserting virtual nodes.^50^ This allows for exploring different postprocessing strategies while leaving the underlying representation and model unchanged. Multiple transforms can also be chained together.

### Model

Models implement neural network architectures and are paired with specific reaction representations. Out-of-the-box, ChemTorch provides representative baselines that reflect common pairings used in the literature, see Table 2. This allows users to benchmark across modalities within a single framework and reuse existing baselines.

**Table 2:** Overview of Neural Network Architectures Implemented in ChemTorch at the Time of Writing

representation	architecture
fingerprint	multilayer perceptron (MLP)
tokenized SMILES	hierarchical attention network (HAN)^38^
graph	D-MPNN,^12,29^ GCN,^51^ GatedGCN,^52^ GAT,^34^ GATv2,^53^ GINE,^54^ GraphGPS,^50^ PNA^55^
3D geometry	DimeNet,^49^ DimeNet++,^30^ DimeReaction^11^

In terms of development workflow, ChemTorch supports both quick prototyping in a standalone model file as well as plug-and-play composition of models from interchangeable submodules. For example, most GNN architectures follow the same blue print and consist of an encoder, multiple message-passing layers, a pooling function, and a prediction head.^56^ ChemTorch takes advantage of this modular composition and implements all GNNs listed in Table 2 under a single modularized interface allowing for systematic exploration and comparison of architectural variants.

### Routine

The routine handles task-specific training logic, including losses, optimizers, schedulers, and metrics. It standardizes how models are trained and evaluated while remaining flexible enough to support custom definitions. ChemTorch currently ships with a general supervised learning routine, which can be used for classification, as well as a dedicated regression routine with optional output scaling to stabilize training when target values vary strongly in magnitude.^12^

### Trainer

The trainer orchestrates the full workflow of training, validation, and testing. By default, ChemTorch relies on the PyTorch Lightning trainer, which ensures reproducibility, efficient logging, and scalability across devices. At the same time, users can integrate custom trainers when specialized optimization protocols are required, for example, when implementing gradient accumulation schemes beyond Lightning’s defaults. This flexibility allows ChemTorch to cover standard use cases while remaining adaptable to advanced experimental setups.

## Features

A central goal of ChemTorch is to make research workflows easier, faster, and more reliable, which is achieved through the following features:

- **Hierarchical configuration:** ChemTorch uses Hydra’s hierarchical configuration system to control all components of a pipeline. Each abstraction (data pipeline, representation, transform, model, routine, trainer) is exposed as a configurable module with sensible defaults. Users can easily mix-and-match components by overriding configuration entries, for example, switching from a fingerprint representation with an MLP to a graph representation with a GNN, without modifying a single line of source code. This design makes experimentation fast, clean, and transparent.
- **Command line interface (CLI):** Experiments can be launched conveniently from a CLI with support for on-the-fly config overrides. Components are instantiated automatically and assembled into a working pipeline, cutting down setup time.
- **Logging, visualization, and run management:** ChemTorch integrates with Weights & Biases for experiment tracking, interactive dashboards, and collaborative sharing of results. Training curves, evaluation metrics, and hardware usage are logged automatically for visualization in real-time. Users can rely on the broad collection of standard metrics provided by TorchMetrics or define custom ones tailored to their specific research questions.
- **Hyperparameter optimization:** ChemTorch enables seamless and resource-efficient hyperparameter optimization through Weights & Biases sweeps. Searches are configured centrally via sweep templates and can use strategies such as random, grid, or Bayesian optimization. Combined with early termination and iteration counts this enables efficient exploration of large parameter spaces.
- **Reproducibility by default:** All resolved configurations are stored together with logs and metrics in Weights & Biases. Thus, any run can be exactly reproduced with a single command from ChemTorch’s CLI.

Together, these features resolve typical bottlenecks in research workflows, enabling faster iteration cycles and making ChemTorch a versatile and user-friendly software platform for model development, hyperparameter optimization, and reproducible benchmarking. For full details, we refer the reader to our official documentation at https://heid-lab.github.io/chemtorch/, which provides a complete API reference, usage tutorials, and a step-by-step guide for getting started.

## Case Study: Barrier-Height Prediction on RDB7

Systematic benchmarks across tasks remain rare, in part because implementations are scattered across task-specific repositories. ChemTorch addresses this gap by providing a unified pipeline for data handling, training, and evaluation, enabling fair cross-modal benchmarks.

Barrier-height prediction offers a compelling testbed for such comparisons, as barrier heights capture the feasibility of a reaction and connect directly to macroscopic observables such as rate constants through transition state theory (TST).^57^ To demonstrate ChemTorch’s benchmarking capabilities, we evaluate four representative architectures from recent literature, covering fingerprint, sequence, graph, and 3D modalities, on the RDB7 data set.^4^ While this initial study is limited to RDB7, future benchmarks should incorporate additional data sets to assess generality across tasks and data regimes.

### Data and Task

The RDB7 data set^4^ contains forward and backward barrier heights (expressed in kcal/mol) as well as transition state geometries for nearly 12,000 organic reactions of unimolecular reactants. Single point energies for these reactions were obtained via QM calculations at CCSD­(T)-F12a/cc-pVDZ-F12//ωB97X-D3/def2-TZVP level of theory. For this study, we restrict ourselves to the forward barriers. The version used in this study can be downloaded from https://github.com/heid-lab/reaction_database.

### Splits and Evaluation

Models were trained and evaluated under several splitting strategies to probe interpolation and extrapolation performance. We used a random 80/10/10 train/validation/test split to test the models’ capability to interpolate in-distribution data. Additionally, we tested extrapolation to out-of-distribution data by evaluating the models on chemically informed data splits: (i) **size**↓/↑: reactions ordered by number of heavy atoms before splitting, simulating extrapolation to smaller/larger systems; (ii) **target**↓/↑: reactions ordered by barrier height, testing extrapolation beyond observed energy ranges; (iii) **reaction core**: reactions grouped by the minimal joint subgraph of reactants and products where bonds are broken or formed, ensuring unseen reaction types in the test set; (iv) **reactant scaffold**: a Bemis–Murcko scaffold split on reactants, as proposed by Spiekermann et al.^11^ Performance was assessed by mean absolute error (MAE) and root mean squared error (RMSE), reported as mean ± standard deviation across random seeds. We used seeds 0–9; however, seed 2 was excluded from all analyses due to a training anomaly in the DimeReaction model, where a single outlier test batch produced a loss of approximately 370 and inflated that run’s RMSE and MAE to 68.46 and 4.45, respectively.

### Models and Tuning

We compared four representative modalities: (1) **DRFP/MLP**, a multilayer perceptron on differential reaction fingerprints;^15^ (2) **SMILES/HAN**, a hierarchical attention network^26,38^ with SMILES syntax-based tokenization;^19^ (3) **CGR/D-MPNN**, a directed message passing neural network^12^ trained on condensed graphs of reaction;^29^ and (4) **DimeReaction**,^11^ a variant of DimeNet++,^30^ which is a 3D message-passing model that predicts reaction barrier heights by passing the difference between TS and reactant (R) geometry embeddings through an additional MLP head. While reactant geometries are relatively inexpensive to obtain, transition state geometries require costly transition state searches and are therefore not readily available. Alternatively, this bottleneck can be partially alleviated by using less costly transition states computed at the semiempirical level, as recently demonstrated by Ferrer et al.^42^ Additionally, recent advances in deep learning have demonstrated first successes in generating approximate TS geometries,^20,21,58^ which can serve as surrogates for the true structures in 3D models such as DimeReaction. In our experiments, we use the ground-truth reactant and TS geometries from RDB7 as oracle features to contextualize the upper bound of achievable accuracy when perfect TS information is available.

Hyperparameters were tuned with Bayesian hyperband^59^ (minimum 30 epochs, η = 1.5) under equal budgets of 100 runs. Sweep ranges included learning rate, weight decay, batch size (32–128), depth, width, number of blocks, cutoff, dropout, and normalization layers where applicable. All models were trained with the AdamW optimizer (β_1_ = 0.9, β_2_ = 0.999), a cosine learning-rate schedule with 10 warmup steps, and mean squared error (MSE) loss. Training employed early stopping (patience 30, Δ = 0.01) and gradient clipping (1.0). Each configuration was repeated with 10 random seeds. All configurations and sweep definitions are available with the ChemTorch code at https://github.com/heid-lab/chemtorch for full reproducibility.

### Compute Environment

All experiments were performed using ChemTorch v0.3.3 (last tested), with full dependency specifications provided in the public repository. Experiments were executed on Rocky Linux 9.5-based machines equipped with NVIDIA A40 GPUs (48 GB memory per GPU, CUDA 12.7), dual-socket AMD EPYC 7413 CPUs (24 cores per socket, 96 logical cores) with each run using a single GPU and 16 GB of system memory. To guard against unintended changes in experimental behavior, ChemTorch includes automated tests designed to detect breaking changes affecting reproducibility.

### Interpolation Performance

The performance of the four modalities on the random split of RDB7 is summarized in Table 3 and Figure 2. Across nine random seeds, graph- and 3D-based models substantially outperform fingerprint- and sequence-based approaches. While DRFP/MLP and SMILES/HAN achieve mean absolute errors (MAEs) of 14.50 and 10.36 kcal/mol, respectively, CGR/D-MPNN and DimeReaction reduce the error to 4.10 and 2.54 kcal/mol, respectively.

**Figure 2:**
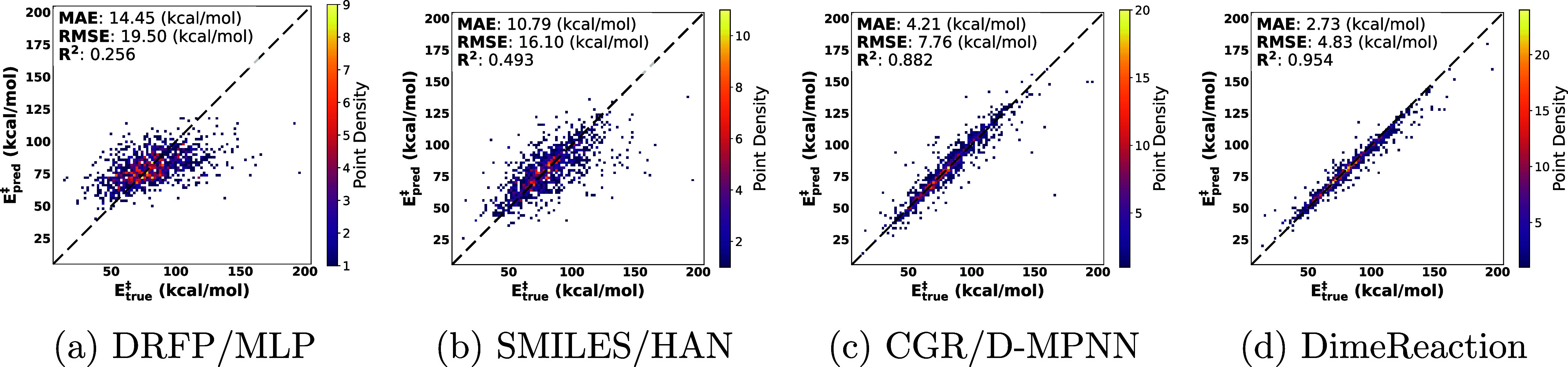
Parity plots showing the performance of (a) DRFP/MLP, (b) SMILES/HAN, (c) CGR/D-MPNN, and (d) DimeReaction on the random split test set (*N*_test_ = 1,194). Note that these are single runs for random seed set to 0. The *x*- and *y*- axis represent the ground truth barrier heights and the models’ predictions, respectively. Individual pixels are colored by the density of data points.

**Table 3:** Comparison of Four Model Classes on RDB7 Forward Reactions^a^

representation	architecture	Params (M)	runtime	MAE
DRFP	MLP	0.6	2 ± 1 min	14.50 ± 0.26
SMILES	HAN	5.0	9 ± 2 min	10.36 ± 0.29
CGR	D-MPNN	4.3	11 ± 2 min	4.10 ± 0.14
R & TS xyz	DimeNet++	2.1	71 ± 1 min	2.54 ± 0.15

a Errors reported as mean ± standard deviation over 9 seeds.

These results highlight three points. First, architectures that explicitly encode chemical structure (graphs or 3D geometries) consistently outperform token- and fingerprint-based baselines by a margin of at least 6 kcal/mol MAE. Second, in line with Spiekermann et al.,^11^ incorporating 3D information markedly improves predictive performance. This makes CGR/D-MPNN a practical and efficient baseline for barrier-height prediction. Third, even when provided with ground-truth reactant and transition state geometries, DimeReaction still exceeds the 1 kcal/mol threshold commonly considered chemical accuracy. This outcome may reflect architectural limitations, residual noise in the RDB7 data set, or the finite size of the training data, which we examine further in the next subsection.

### Scaling and Data Efficiency

To study how performance scales with data set size, we retrained each model on progressively smaller subsets of the training data, which can be easily done via the ChemTorch CLI by specifying the subsample options. For this analysis, we retrained only one model per data set size using seed 0.

Across all architectures, the learning curves in Figure 3 show continued improvement toward the right-hand side of the plot, with no clear evidence of saturation at the largest data set sizes. On the logarithmic *x*-axis, their approximately linear behavior indicates that increasing the training set by an order of magnitude yields a comparable reduction in error. This suggests that all models would still benefit from additional data, although the absolute gains at the current error level (≈2.5 kcal/mol) are unlikely to match the ∼5–7 kcal/mol reduction observed for SMILES/HAN, CGR/D-MPNN, and DimeReaction when increasing the training set from 1000 to 10,000 reactions.

**Figure 3:**
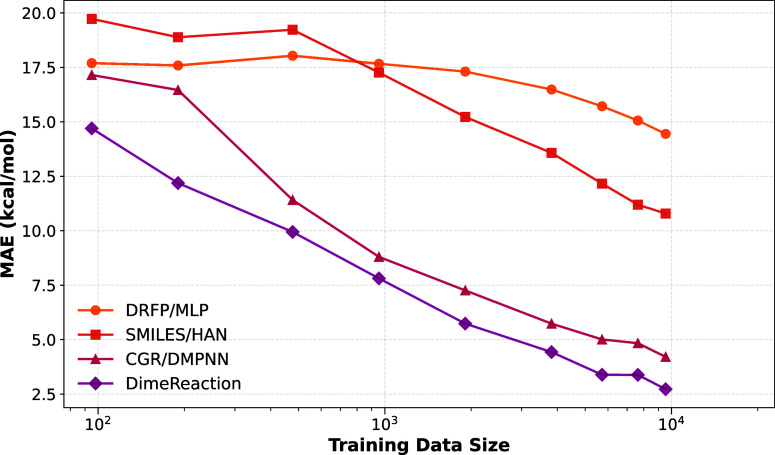
Mean absolute error as a function of training set size on RDB7. Graph- and 3D-based models not only reach lower error but also exhibit steeper learning curves, highlighting their superior data efficiency.

Beyond overall scaling behavior, performance curves (Figure 3) reveal that CGR/D-MPNN and DimeReaction consistently outperform SMILES/HAN and DRFP/MLP across all training sizes. Notably, they reach a lower error with fewer than 1000 reactions (one tenth of the training data) than DRFP/MLP and SMILES/HAN using the full data set. The learning curve of SMILES/HAN exhibits a similar slope to CGR/D-MPNN and DimeReaction, indicating comparable data-efficiency despite a higher baseline error. In contrast, fingerprint-based DRFP/MLP improves more slowly and remains markedly less accurate throughout. These results suggest that learned representations capture transferable reaction patterns more effectively than fingerprint-based descriptors, a key consideration given that reaction data sets often comprise only a few thousand examples.

### Out-of-Distribution Generalization

In practice, a model’s usefulness depends on its ability to generalize to unseen data. While random train–validation-test splits are commonly used in evaluation of machine learning models, they primarily assess the model’s ability to interpolate within the training distribution. Real-world reaction modeling tasks, however, routinely involve out-of-distribution (OOD) scenarios, such as larger molecules than those seen in training, reactions with higher or lower barriers, or entirely new reaction types. Indeed, Vadaddi et al.^9^ find that state-of-the-art GNNs can approach irreducible errors on in-distribution data but fail catastrophically under OOD evaluation. These observations motivate the use of multiple data splitting strategies designed to probe distinct notions of generalization.

To assess generalization beyond random splits, we evaluated all models under a range of chemically motivated out-of-distribution (OOD) settings using the data splitters implemented in ChemTorch (Table 4). The resulting performance profiles are summarized in Figure 4. When normalizing the MAE on each split by the best-performing model the relative ranking of models remains consistent across all settings, with DimeReaction outperforming CGR/D-MPNN, followed by SMILES/HAN and DRFP/MLP (Figure 4, left). This stability suggests that architectural inductive biases beneficial for in-distribution performance largely transfer across different OOD scenarios. Normalizing each model’s MAE by its own performance on the random split reveals split-specific generalization gaps (Figure 4, right). All models interpolate reliably to smaller molecules, with GNN-based approaches even improving relative to the random baseline, whereas extrapolation to larger molecules leads to systematic performance degradation. The most pronounced failure occurs under target-based splits, where all models exhibit severe error increases when predicting barrier heights outside the range observed during training, highlighting the fundamentally interpolative nature of neural network models.

**Figure 4:**
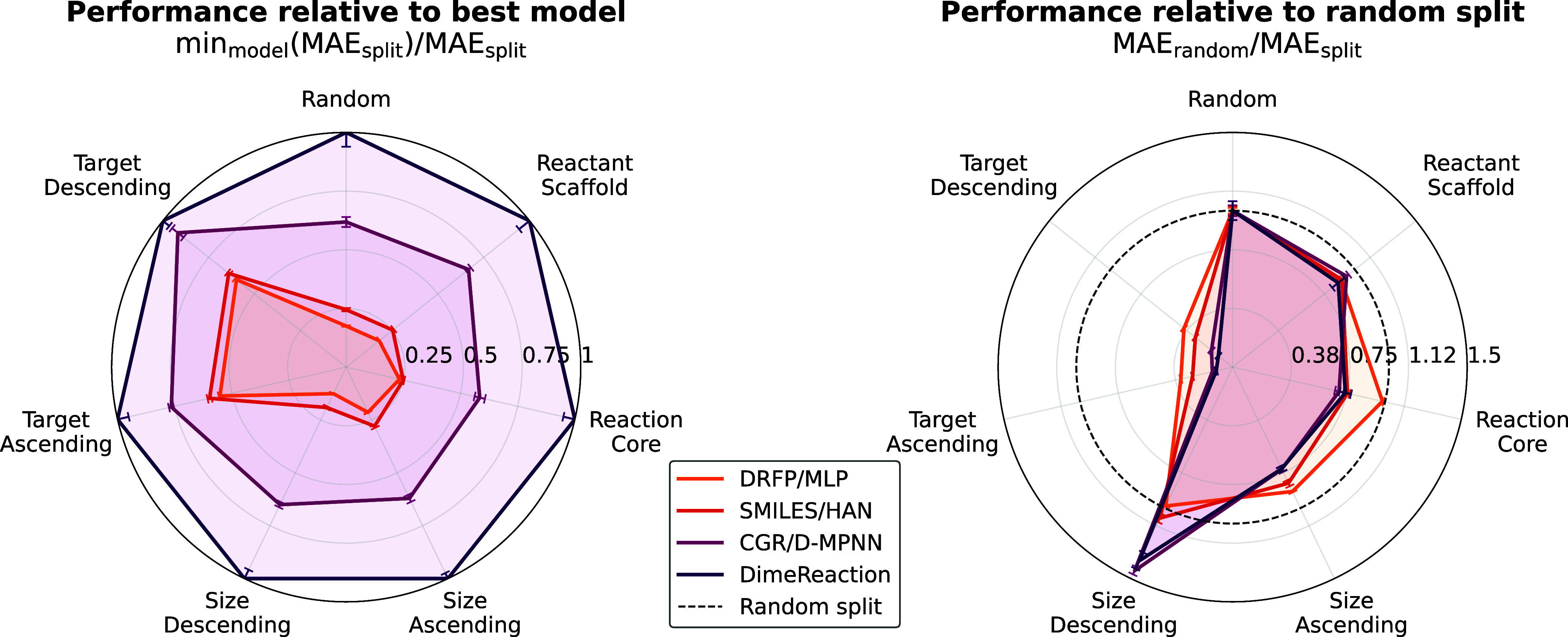
Comparison of OOD performance (higher is better); mean ± standard deviation over 9 seeds. Left: radar plot showing the MAE of each model scaled by the MAE of the best-performing model on the respective split. Right: radar plot displaying each model’s MAE scaled by to the same model’s MAE on the random split.

**Table 4:** Test Set Performance across Different Data Splitting Strategies^a^

split	model	MAE	RMSE
random	DRFP/MLP	14.50 ± 0.26	19.06 ± 0.36
	SMILES/HAN	10.36 ± 0.29	15.01 ± 0.58
	CGR/D-MPNN	4.10 ± 0.14	7.25 ± 0.35
	DimeReaction	2.54 ± 0.15	4.45 ± 0.38
reactant scaffold	DRFP/MLP	16.30 ± 0.17	21.19 ± 0.26
	SMILES/HAN	11.59 ± 0.26	16.03 ± 0.35
	CGR/D-MPNN	4.40 ± 0.08	7.31 ± 0.10
	DimeReaction	2.94 ± 0.17	4.77 ± 0.19
reaction core	DRFP/MLP	14.75 ± 0.11	18.89 ± 0.14
	SMILES/HAN	13.75 ± 0.25	18.31 ± 0.35
	CGR/D-MPNN	5.88 ± 0.23	8.69 ± 0.23
	DimeReaction	3.43 ± 0.17	5.09 ± 0.18
size descending	DRFP/MLP	14.72 ± 0.07	18.96 ± 0.07
	SMILES/HAN	9.68 ± 0.26	14.15 ± 0.27
	CGR/D-MPNN	2.84 ± 0.05	5.62 ± 0.13
	DimeReaction	1.85 ± 0.08	4.05 ± 0.19
size ascending	DRFP/MLP	16.44 ± 0.11	21.57 ± 0.18
	SMILES/HAN	12.59 ± 0.31	17.88 ± 0.37
	CGR/D-MPNN	5.67 ± 0.15	9.54 ± 0.22
	DimeReaction	3.52 ± 0.09	6.15 ± 0.29
target descending	DRFP/MLP	36.42 ± 0.45	37.95 ± 0.47
	SMILES/HAN	34.01 ± 0.34	36.22 ± 0.47
	CGR/D-MPNN	23.73 ± 0.81	25.41 ± 0.81
	DimeReaction	21.79 ± 0.77	23.55 ± 1.03
target ascending	DRFP/MLP	42.71 ± 0.42	45.06 ± 0.41
	SMILES/HAN	39.46 ± 0.57	42.20 ± 0.51
	CGR/D-MPNN	30.85 ± 0.42	33.58 ± 0.46
	DimeReaction	23.59 ± 1.15	26.17 ± 1.15

a Errors reported as mean ± standard deviation over 9 seeds.

In general, data splits that explicitly partition reactions by chemical structure such as reactant-scaffold and reaction-core splits lead to a marked degradation in performance across models, reflecting the increased difficulty of generalizing to structurally or mechanistically novel reactions. The sole exception is DRFP/MLP, which exhibits almost no relative performance degradation on the reaction-core split. However, this apparent robustness reflects a representational limitation rather than a strength of the model. By design, DRFP captures which local environments appear or disappear across a reaction, but not how they are transformed within a specific mechanism.^15^ Consequently, DRFP/MLP cannot exploit the mechanistic information removed by reaction-core splitting, which may also explain its comparatively weak overall performance. In contrast, the reactant-scaffold split alters the distribution of substructures present in the input reactions, directly affecting the fingerprint representation and leading to a measurable drop in DRFP/MLP performance.

For models that can leverage structural and mechanistic information, the reactant-scaffold split introduced by Spiekermann et al.^11^ yields errors comparable to the random baseline. This result is misleading, as products in the test set may share scaffolds with reactants in the training set, leading to “scaffold” leakage between partitions. As a consequence, scaffold-based splits can substantially overestimate a model’s ability to generalize to novel reaction chemistry. For chemical reaction modeling, we therefore recommend using stricter schemes such as reaction-core splits, which better isolate mechanistic novelty and provide a more realistic assessment of out-of-distribution performance. More fine-grained splits based on specific chemical motifs or functional groups represent a complementary direction for future benchmarks and can be readily incorporated within the ChemTorch framework.

Taken together, these results demonstrate that conclusions about model generalization depend critically on the chosen evaluation protocol, as well as on the types of information a given neural network architecture can exploit. These factors largely determine a model’s suitability for a particular task. By standardizing and comparing multiple data splitting strategies within a single framework, ChemTorch enables a more nuanced and realistic assessment of model robustness, helping to avoid overly optimistic conclusions drawn from random splits alone.

## Conclusion

We introduced ChemTorch, a modular and extensible open-source framework that supports diverse chemical reaction representations in a single code base. By abstracting data pipelines, representations, models, and training routines into interchangeable components, ChemTorch drastically reduces the engineering overhead associated with developing novel reaction representations and neural network architectures.

As a case study, we applied ChemTorch to RDB7 barrier-height prediction using fingerprint, sequence, graph, and 3D-based models. This comparison illustrates how ChemTorch can be used to conduct cross-modal benchmarks with minimal effort. Our results reproduce trends reported in prior work. As noted by Spiekermann et al.,^11^ GNNs like D-MPNNs trained on the CGR provide a computationally efficient baseline that only requires atom-mapping of reactant and product graphs. When reactant and transition state structures are known, 3D models like DimeReaction deliver the highest accuracy. Similar to Vadaddi et al.,^9^ we observe that model generalization can be highly sensitive to the choice of evaluation protocol. Specifically, our results show that all architectures fail to generalize in multiple chemically relevant OOD scenarios to varying extents. In general, we observe that learning directly on structural information such as molecular graphs or 3D geometries leads to a better inductive bias. However, different architectures and training approaches may yield different performance levels. In particular, transformer-based sequence models such as BERT variants may be more expressive than HANs, and unsupervised pretraining on large reaction corpora may boost the performance of sequence-based approaches.^16,60^ In any case, this highlights the need for further investigation and broader benchmarks, for which ChemTorch provides a convenient platform.

Looking ahead, ChemTorch establishes a foundation for systematic exploration of reaction learning problems across modalities and tasks. Its design makes it straightforward to integrate new data sets, models, and training protocols, while its built-in splitters and hyperparameter optimization tools facilitate transparent and reproducible research. We envision ChemTorch evolving into a community-driven platform, where shared data sets and leaderboards motivate collective progress. As an initial step toward this vision, we provide a centralized public repository hosting commonly used quantum-mechanical reaction data sets and support community contributions via a documented, pull request-based workflow. Beyond academic benchmarks, such a framework can accelerate the translation of reaction modeling to practical applications in synthesis planning, catalysis, and automated discovery.

## Data Availability

The data used in this study can be downloaded from GitHub: https://github.com/heid-lab/reaction_database. ChemTorch’s source code is publicly available on GitHub: https://github.com/heid-lab/chemtorch.

## Footnotes

1
https://github.com/heid-lab/reaction_database; data set contributions are welcome (see repository documentation)
